# Comparing the pooled cohort equations and coronary artery calcium scores in a symptomatic mixed Asian cohort

**DOI:** 10.3389/fcvm.2023.1059839

**Published:** 2023-01-17

**Authors:** Lohendran Baskaran, Jing Kai Lee, Michelle Shi Min Ko, Subhi J. Al’Aref, Yu Pei Neo, Jien Sze Ho, Weiting Huang, Yeonyee Elizabeth Yoon, Donghee Han, Rine Nakanishi, Swee Yaw Tan, Mouaz Al-Mallah, Matthew J. Budoff, Leslee J. Shaw

**Affiliations:** ^1^Department of Cardiology, National Heart Centre Singapore, Singapore, Singapore; ^2^Duke-NUS Medical School, National University of Singapore, Singapore, Singapore; ^3^Division of Cardiology, Department of Medicine, University of Arkansas for Medical Sciences, Little Rock, AR, United States; ^4^Seoul National University Bundang Hospital, Seongnam-si, Republic of Korea; ^5^Department of Imaging, Cedars-Sinai Medical Center, Los Angeles, CA, United States; ^6^Department of Cardiovascular Medicine, Toho University Graduate School of Medicine, Tokyo, Japan; ^7^Houston Methodist DeBakey Heart and Vascular Center, Houston Methodist Hospital, Houston, TX, United States; ^8^Department of Medicine, Lundquist Institute at Harbor-UCLA Medical Center, Torrance, CA, United States; ^9^Icahn School of Medicine at Mount Sinai, Blavatnik Family Women’s Health Research Institute, New York, NY, United States

**Keywords:** pooled cohort equation, coronary artery calcium score, Agatston score, major adverse cardiovascular events, predictive model

## Abstract

**Background:**

The value of pooled cohort equations (PCE) as a predictor of major adverse cardiovascular events (MACE) is poorly established among symptomatic patients. Coronary artery calcium (CAC) assessment further improves risk prediction, but non-Western studies are lacking. This study aims to compare PCE and CAC scores within a symptomatic mixed Asian cohort, and to evaluate the incremental value of CAC in predicting MACE, as well as in subgroups based on statin use.

**Methods:**

Consecutive patients with stable chest pain who underwent cardiac computed tomography were recruited. Logistic regression was performed to determine the association between risk factors and MACE. Cohort and statin-use subgroup comparisons were done for PCE against Agatston score in predicting MACE.

**Results:**

Of 501 patients included, mean (SD) age was 53.7 (10.8) years, mean follow-up period was 4.64 (0.66) years, 43.5% were female, 48.3% used statins, and 50.0% had no CAC. MI occurred in 8 subjects while 9 subjects underwent revascularization. In the general cohort, age, presence of CAC, and ln(Volume) (OR = 1.05, 7.95, and 1.44, respectively) as well as age and PCE score for the CAC = 0 subgroup (OR = 1.16 and 2.24, respectively), were significantly associated with MACE. None of the risk factors were significantly associated with MACE in the CAC > 0 subgroup. Overall, the PCE, Agatston, and their combination obtained an area under the receiver operating characteristic curve (AUC) of 0.501, 0.662, and 0.661, respectively. Separately, the AUC of PCE, Agatston, and their combination for statin non-users were 0.679, 0.753, and 0.734, while that for statin-users were 0.585, 0.615, and 0.631, respectively. Only the performance of PCE alone was statistically significant (*p* = 0.025) when compared between statin-users (0.507) and non-users (0.783).

**Conclusion:**

In a symptomatic mixed Asian cohort, age, presence of CAC, and ln(Volume) were independently associated with MACE for the overall subgroup, age and PCE score for the CAC = 0 subgroup, and no risk factor for the CAC > 0 subgroup. Whilst the PCE performance deteriorated in statin versus non-statin users, the Agatston score performed consistently in both groups.

## 1. Introduction

Atherosclerotic cardiovascular disease (ASCVD) is the largest cause of death globally (1, 2). To aid risk assessment in symptomless patients, numerous risk scores have been developed, including the guideline recommended pooled cohort equations (PCE), which calculate a patient’s cardiovascular risk based on various established risk factors (3–6).

Concurrently, the assessment of coronary artery calcium (CAC) using computed tomography (CT) has also emerged as a powerful prognostic tool (7–9). CAC quantification using the Agatston scoring method has shown superior risk prediction to other serum and imaging biomarkers (10–13). A zero CAC score serves as a good prognostic marker against cardiovascular mortality in symptomless individuals with otherwise low to moderate risk (14). In subjects with non-zero CAC, various studies have demonstrated an association between an increased CAC and risk of MACE (7, 11, 15–19). This has resulted in its inclusion in guidelines in select individuals (5, 20, 21). Although the incorporation of CAC has been shown to improve risk assessment over and above PCE in asymptomatic patients, its utility in stable chest pain patients is less well established (22, 23).

Additionally, the prevalence and implications of ASCVD and CAC burden in Asian populations is less well understood. Studies in Asian populations demonstrate an extension of the 15-year warranty rule in some cohorts (24), but not others (25), suggesting that the utility of risk assessment tools designed for one population may be blunted in others (26–30). Further, while studies on an Asian cohort have identified various risk factors for CAC progression and their use as a predictive tool for progression, those applicable to the Southeastern Asia context are lacking (31, 32). Secondarily, although prior studies have demonstrated the pro-calcific effect of statins on atherosclerotic plaque (33–35), this has not been studied in a Southeast Asian context. Singapore’s population comprises three major Asian ethnicities [Chinese (74.3%), Malay (13.5%), and Indian (9.0%)], with genetic diversity representing East Asia, Southeast Asia, and South Asia (36, 37), respectively.

We sought to investigate the predictive value of CAC amongst a symptomatic ethnically diverse Asian cohort and examine the association between these risk prediction tools and major cardiovascular events (MACE). We also sought to evaluate these scores in subgroups based on statin use. We hypothesized that within a symptomatic cohort in Singapore – a population whom CAC score is not routinely used for risk stratification – CAC provides incremental performance over PCE in the prediction of MACE.

## 2. Materials and methods

### 2.1. Study participants

**FIGURE 1:**
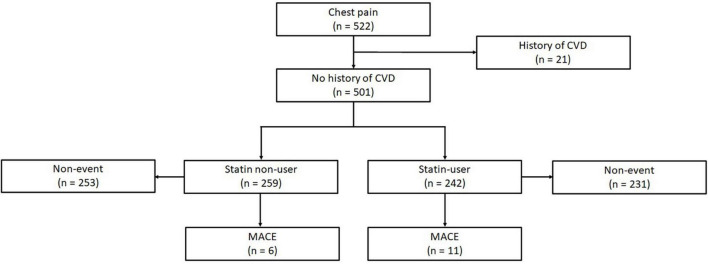
Of 522 subjects with complete risk factor profiles, 21 participants were first excluded for prior history of ASCVD. 501 subjects were suitable for analysis, amongst which 259 were statin non-users with 6 events, and 242 were statin-users with 11 events. CVD, cardiovascular disease; CAC, coronary artery calcium; MACE, major adverse cardiovascular events.

This was a retrospective cohort study from a registry of individuals who had undergone cardiac CT between November 2010 to October 2017 at a tertiary cardiac institution in Singapore, a city-state comprising three major Asian ethnicities that broadly represent large parts of Asia—Chinese, Malay, Indian, and other ethnicities. Subjects who underwent clinically indicated CT scans for symptomatic suspected CAD from July 2015 to October 2017 were eligible. Patients aged 21 and above with complete risk factor profile (*n* = 522) were included (Figure 1). 21 patients with known prior history of myocardial infarction (MI), heart failure, or revascularization procedures such as percutaneous coronary intervention (PCI) or coronary artery bypass graft (CABG) were excluded. The final number of subjects included in this study was 501. No images were of poor quality and there was no missing data for this final cohort. The use of patient data for this study was approved by, and in accordance to, the guidelines and regulations of SingHealth Centralised Institutional Review Board (CIRB). Waiver of informed consent has been approved by CIRB.

### 2.2. CT acquisition and CAC quantification

Non-contrast gated cardiac CAC scans were performed using a 320 slice multi-detector CT scanner (Toshiba Aquilion ONE) in accordance with the Society of Cardiovascular CT (SCCT) guidelines (38). CAC images were acquired using 120 kVp, 300–600 mAs, prospective ECG gating, and 3 mm reconstructions.

Coronary artery calcium scoring was first calculated using the Agatston quantification method (10). The CAC volume score was computed as previously defined by Callister et al. (39). In brief, it is the sum of the total volume of calcium of the calcified regions in all vessels multiplied by 1000.

### 2.3. Ascertainment of ASCVD risk factor status and risk scoring

Risk factors and symptom status was verified in two ways: using a survey questionnaire filled in the CT lab and through electronic healthcare records comprising physician diagnosis in clinic and lab results. Laboratory measurements such as total cholesterol (TC), high-density-lipoprotein-cholesterol (HDL-C), and low-density lipoprotein-cholesterol (LDL-C) levels only included results that were within 1 year of CAC CT scan acquisition. Family history of premature CAD was defined as a first-degree male relative of age <55 years or a first-degree female relative of age <65 years. Former and current smokers constituted a positive smoking history. Hypercholesterolemia was defined as total serum cholesterol of greater than 5.5 mmol/L, or if the patient was on statin therapy. Diabetic status was confirmed on any one of: (1) fasting plasma glucose ≥ 7 mmol/L (126 mg/dL); (2) glycated hemoglobin ≥ 6.5%; (3) an existing physician diagnosis or; (4) were on diabetic medication. Patients were deemed hypertensive if they had any one of: (1) systolic blood pressure ≥ 140 mmHg; (2) diastolic blood pressure ≥ 90 mmHg; (3) an existing physician diagnosis or; (4) were on anti-hypertensive medication. Chest pain was defined as typical, atypical, or non-anginal. Typical chest pain was defined as: (1) substernal pain or discomfort; that was (2) provoked by exertion or emotional stress; and (3) relieved by rest or nitrate. Atypical chest pain was defined as 2 of the previously mentioned criteria. If 1 or none of the criteria was present, chest pain was categorized as non-anginal (40, 41). Dyspnea on exertion was considered as equivalent to typical chest pain. An ASCVD risk score was then calculated using the PCE, that was originally derived and validated using multiple US-based prospective studies. The PCE generates a score based on the ATP III model using patient risk factors. These include age, gender, smoking status, total cholesterol, HDL-C, and LDL-C levels, systolic blood pressure, and blood pressure treatment. The ATP III model was chosen as it does not require country-specific ethnicity and it assess for similar endpoints (3).

### 2.4. Outcome determination

Outcomes were determined by assessing the patient’s electronic medical records. Records that were reviewed include the clinical notes entered for all inpatient, outpatient, and emergency department visits, as well as a report of investigations that were performed. All available records were reviewed, starting from the initial consult until the last available consult, discharge from follow-up, or until the intended endpoint was met. The primary endpoint was MACE, defined as the composite of cardiac death, MI, hospitalization due to heart failure, and revascularization. Non-fatal MI and cardiac death were defined using clinical symptoms, signs on ECG, increased cardiac enzymes. Revascularization was defined as PCI or CABG that occurred beyond 180 days of the index scan.

### 2.5. Statistical analysis

In all analyses, both CAC volume and Agatston score were transformed with the natural logarithm (ln) as previous studies have found a linear relation between the natural logarithmic forms and the risk of MACE (8). Univariate logistic regression analysis was then performed for ln(Agatston) and ln(Volume), as well as the ASCVD risk factors, for all primary and secondary endpoints for the total population, as well as for CAC-zero and CAC non-zero subgroups. For the risk factors, age, gender, smoking status, systolic blood pressure, blood pressure treatment, total cholesterol, HDL-C, as well as total PCE score were analyzed.

From the results, a multivariate logistic regression analysis was performed for our variable of interest, ln(Agatston), adjusted for age. Area under the receiver operating characteristic curve (AUC) analyses were then performed to assess the discriminative performance of the PCE score and ln(Agatston) score, as well as the incremental values of each. SPSS Statistic version 25 and MedCalc version 20.011 was used for all statistical analyses in this study.

## 3. Results

### 3.1. Clinical characteristics

A total of 501 subjects met the criteria and were included in this study, with a mean age of 53.7 ± 10.8 years, and 43.5% were female (Table 1). 251 (50.1%) had a zero CAC. Ethnically, 76.3% were Chinese, 5.1% Malay, 8.7% Indian, and 9.9% other Asian ethnicities, including Eurasian, Indonesian, Bangladeshi, and Filipino. Subjects with higher Agatston scores were more likely to be male, older, diabetic, hypertensive, and have higher CAC volume. Of the cohort, statin was used in 48.3%.

**TABLE 1:** Clinical characteristics of patients stratified across coronary artery calcium (CAC) Agatston score categories.

	CAC = 0 (*n* = 251)	CAC = 1–10 (*n* = 44)	CAC = 11–100 (*n* = 94)	CAC = 101–400 (*n* = 66)	CAC > 400 (*n* = 46)	All (*n* = 501)	*P*
Age, years	49.4 ± 9.72	55.8 ± 8.5	55.7 ± 9.8	60.2 ± 8.8	60.9 ± 12.4	53.7 ± 10.8	<0.01
Female (%)	51.0	52.3	55.6	30.3	21.7	43.5	<0.01
Smoker (%)	23.5	11.4	12.1	30.3	17.4	23.4	0.15
Diabetic (%)	8.4	15.9	16.9	24.2	21.7	14.0	<0.01
Hypertension (%)	28.7	40.9	43.5	50.0	69.6	40.1	<0.01
Statin use (%)	34.7	56.8	56.4	69.7	67.4	48.3	<0.01
Total cholesterol, mmol/L	5.1 ± 1.1	5.1 ± 1.0	5.1 ± 1.1	4.8 ± 1.3	4.4 ± 0.9	5.0 ± 1.1	<0.01
HDL-C, mmol/L	1.3 ± 0.4	1.3 ± 0.3	1.3 ± 0.3	1.2 ± 0.3	1.3 ± 0.3	1.3 ± 0.3	0.19
LDL-C, mmol/L	3.3 ± 2.3	3.1 ± 0.9	3.0 ± 0.9	2.9 ± 1.1	2.5 ± 0.8	3.1 ± 1.7	<0.01
PCE risk score	11.0 ± 4.5	12.7 ± 3.8	13.0 ± 3.8	14.4 ± 3.4	13.8 ± 4.7	12.4 ± 4.4	<0.01
CAC volume, mm^3^	0 ± 0	7.5 ± 4.2	47.6 ± 25.4	203.3 ± 77.6	826.0 ± 455.9	113.6 ± 276.4	<0.01
CAC density	0 ± 0	1.5 ± 0.5	2.9 ± 0.7	3.3 ± 0.4	3.5 ± 0.3	1.4 ± 1.5	<0.01
**Chest pain (%)**							
Non-anginal	31.5	34.1	30.9	25.8	32.6	30.9	
Atypical	33.5	31.8	31.9	31.8	26.1	32.1	
Typical	35.1	34.1	37.2	42.4	41.3	36.9	

*P*-value represents the significance between the mean or proportion across the CAC 0, 1–10, 11–100, 101–400, and >400 subgroups.

### 3.2. Association of ASCVD risk factors and CAC scores to events

Mean follow-up period was 4.64 ± 0.66 years, during which there were 17 MACE. MI occurred in eight subjects while nine subjects underwent revascularization. Of these, only one MI was in a zero CAC subject.

Of the CAC scores, univariate analysis showed ln(Volume) CAC to be associated with MACE (Table 2). Ln(Agatston) trended toward but was not significant (*p* = 0.056). CAC density was not significant (*p* = 0.263). The presence of CAC confers an OR of 7.95 for MACE (*p* = 0.008). ASCVD risk factors, either separately or aggregated into the PCE (*p* = 0.135) were not associated with MACE. The multivariate model was performed for ln(Agatston), adjusted for age, which was significant in the univariate analysis. Following adjustment, OR of ln(Agatston) was >1, but was not statistically significant (*p* = 0.057). When segregated into subgroups (Table 3), age (OR = 1.16, *p* = 0.028) and PCE (OR = 2.42, *p* = 0.024) score were significantly associated with MACE in the CAC = 0 subgroup, but not the CAC > 0 subgroup. None of the CAC parameters or the ASCVD risk factors were significantly associated with MACE in the CAC > 0 subgroup.

**TABLE 2:** Univariate and multivariate analysis for CAC scores and various risk factors for MACE.

Univariate	Odds ratio	*P*
Age	1.05 (1.01–1.10)	0.026
Male	2.58 (0.83–8.01)	0.102
Smoker	1.01 (0.32–3.16)	0.986
Hypertension	2.19 (0.82–5.86)	0.118
Total cholesterol, per mmol/L	0.78 (0.49–1.25)	0.298
HDL-C, per mmol/L	0.29 (0.06–1.52)	0.145
PCE score	1.10 (0.97–1.25)	0.135
+ve CAC	7.95 (1.80–35.1)	0.008
CAC density	1.49 (0.74–3.02)	0.263
Ln(Agatston)	1.36 (0.99–1.86)	0.056
Ln(Volume)	1.44 (1.01–2.05)	0.044
**Multivariate**		
Age	1.00 (0.95–1.05)	0.851
ln(Agatston)	1.38 (0.99–1.91)	0.057

**TABLE 3:** Subgroup univariate analysis between CAC = 0 and CAC > 0 for CAC scores and various risk factors for MACE.

Univariate	Odds ratio (CAC = 0)	*P*	Odds ratio (CAC > 0)	*P*
Age	1.16 (1.02–1. 32)	0.028	1.01 (0.96–1.06)	0.753
Male	0.96 (0.06–15.5)	0.977	0.43 (0.12–1.55)	0.195
Smoker	0.30 (0.02–4.93)	0.402	1.22 (0.33–4.49)	0.762
Hypertension	–	–	0.93 (0.33–2.64)	0.890
Total cholesterol, per mmol/L	1.24 (0.38–4.09)	0.725	0.78 (0.47–1.30)	0.339
HDL-C, per mmol/L	2.23 (0.07–72.1)	0.650	0.18 (0.03–1.31)	0.090
PCE score	2.42 (1.13–5.20)	0.024	0.99 (0.86–1.13)	0.843

Odds ratio for hypertension in the CAC = 0 subgroup is not available as all participants with MACE had hypertension.

### 3.3. Discriminatory performance of PCE and CAC scores for predicting MACE

**FIGURE 2:**
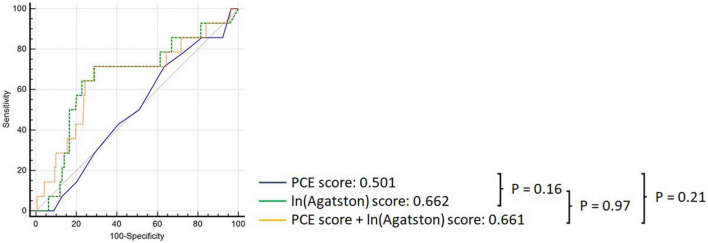
Comparison of ROC curves demonstrating discrimination of the PCE (black), Agatston score (green), and PCE + Agatston score (yellow) models in predicting MACE in the overall cohort.

**FIGURE 3:**
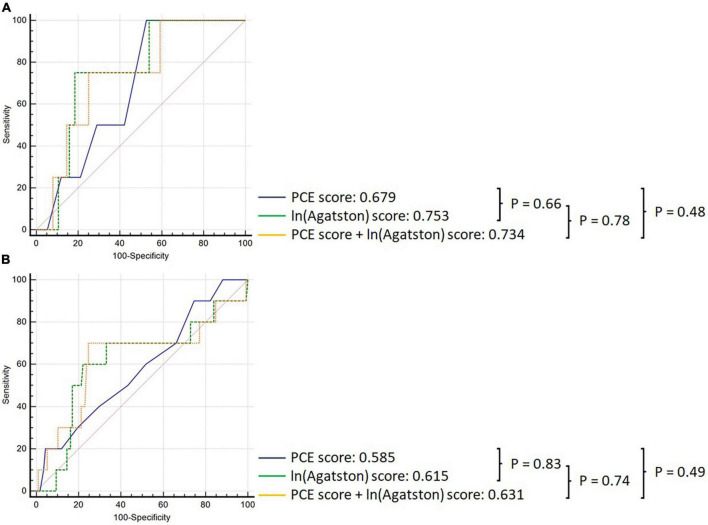
Comparison of ROC curves demonstrating discrimination of the PCE (black), Agatston score (green), and PCE + Agatston score (yellow) models in predicting MACE, in panel **(A)** non-statin-prescribed participants, and **(B)** in statin-prescribed participants.

In the overall cohort, the PCE obtained an AUC of 0.501 (95% CI: 0.432–0.571), and the Agatston score alone obtained an AUC of 0.662 (95% CI: 0.593–0.726); however, this difference was not statistically significant *p* = 0.164 (Figure 2). Adding the Agatston score did not improve discrimination above the PCE [AUC 0.662, 95% CI: 0.593–0.725 (*p* = 0.205)]. This pattern of discriminative performance remained undifferentiated in the statin or non-statin user subgroups (Figure 3).

### 3.4. Statin use and score performance

Pooled cohort equations alone performed better in the statin non-user group (AUC 0.783 95%CI: 0.715–0.841) than in the statin user group [AUC 0.507, 95% CI: 0.434–0.580], (*p* = 0.025). Conversely, the Agatston score maintained consistent discriminatory performance in statin users [AUC 0.707 (95% CI: 0.646–0.764)] vs statin non-users [AUC 0.825 (95% CI: 0.773–0.869), (*p* = 0.369)]. The incremental discriminatory performance of the Agatston score added to the PCE was not significantly different in statin non-users [AUC 0.734, 95% CI: 0.623–0.826) versus statin users [AUC 0.630, 95% CI: 0.540–0.714 (*p* = 0.533)].

## 4. Discussion

In this study using a symptomatic mixed Asian cohort, the Agatston score did not provide incremental discriminatory performance for MACE than the PCE in the overall group. The PCE performed better in the non-statin user subgroup compared to statin users, whereas the Agatston score performed equally well in both non-statin and statin users. This is the first study evaluating these methods in a symptomatic singular mixed cohort comprising East Asian, Southeast Asian, and South Asian ethnicities.

In this study, the PCE and its ASCVD risk factor components were not associated with MACE. Furthermore, the PCE had poor discriminatory performance. In a prior study evaluating the PCE in asymptomatic subjects from the CAC Consortium by Blaha et al. (23) the PCE obtained a higher AUC of 0.79. The disconnect between that study and the current one could be explained by two factors.

Firstly, the current study only included symptomatic patients, whereas the PCE was originally developed for use in asymptomatic patients. As such, the risk factor profile of the studied cohorts may vary substantially. In the study by Blaha et al. (23) the overall 10-year risk was 8.1%, whereas in the current study it was 13.5%. A MESA substudy of asymptomatic smokers with a more comparable overall risk of 14.1% also obtained a lower AUC of 0.69 (42).

Second, as symptomatic patients presumably have a larger burden of disease, most of the MACE events were driven by revascularization initiated by stable chest pain symptoms. This is as opposed to MI or cardiovascular death in asymptomatic patients, where the culprit lesions are usually non-obstructive (43).

The PCE performed better in the statin non-user group compared to the statin user group. This attenuated performance of the PCE amongst statin users is in concordance with prior studies. In a prior contemporary “real-world” registry, the PCE overestimated MACE by more than five times in statin users across the risk category spectrum, with resultant AUCs of 0.6 to 0.7 (44). In a separate study evaluating the PCE using a large national health care system cohort, baseline statin use was associated with an 8% lower MACE risk (45).

Although originally developed for use in asymptomatic subjects, the PCE combined with the Agatston score have been evaluated in cohorts with stable chest pain. In another study involving symptomatic patients referred for stress positron emission tomography (PET) testing, the Agatston score obtained an AUC of 0.65 vs 0.57 for the PCE, and revascularization comprised 73% of all MACE (22). The improved utility of CAC was also seen when compared to other imaging methods. In the largest study of symptomatic patients to date, Budoff et al. (46) examined the prognostic value of CAC versus functional testing (FT) in 8,811 patients from the Prospective Multicenter Imaging Study for Evaluation of Chest Pain (PROMISE) trial. In that study, CAC assessment improved the AUC for prediction of cardiovascular mortality, myocardial infarction, and unstable angina from 0.52 to 0.58. These findings have been reproduced in asymptomatic subjects. In the Heinz Nixdorf Recall (HNR) study, the Agatston score obtained an AUC of 0.74 vs 0.68 for the Framingham Risk Score (FRS) (7).

Coronary artery calcium and its quantification *via* the Agatston score reveal the result of all exposomes toward the pathological process, rather than indirectly using a limited number of contributary risk factors as done by the PCE, thus accounting for its superior predictive performance. Discrepancies between the PCE and the Agatston score have previously been demonstrated, suggesting that >40% of subjects may benefit from additional CAC testing to clarify risk (47).

In the current study, the Agatston score trended toward a higher AUC when added to the PCE in both statins and non-user cohort. However, this was not statistically significant. Although statin use has been associated with increased plaque calcification and reduced MACE in those with existing CAC, the current study does not support any differentiation in the discriminatory performance of CAC score in statin versus non-statin users (48, 49). This may be due to the so-called CAC paradox in this current cohort the majority of statin users may explain these findings (50). The incremental performance of the Agatston score has been more marked in studies with a lower proportion of subjects on lipid-lowering therapies. In the analysis of the CAC Consortium by Blaha et al. (23) adding the Agatston score improved the AUC from 0.79 to 0.82. In that study, the incremental performance of CAC assessment to PCE was seen in both statin users and non-users, which follows the same trend as the current findings. The current study may therefore have been underpowered to reproduce the full differences seen in other studies.

Instead, numerous studies have shown other measures of CAC to be significant markers of MACE over and above the Agatston score, including diffusivity, lesion size, number of vessels and location (9, 51–53). Machine learning methods to identify, quantify and analyze CAC on a pixel-to-whole heart scale may aid in providing more refined methods (54, 55).

The results from this study may have implications in the management of symptomatic patients. They suggest that using the PCE alone for risk assessment may not be sufficient or indeed necessary. Rather, it suggests that in this higher-risk profile group, evaluating CAC using the Agatston score either alone or in tandem may provide value in risk clarification. However, the benefit of either CAC evaluation or PCE assessment is attenuated in statin users. Whilst prior studies and guidelines suggest CAC evaluation in similar intermediate (≥7.5%) risk patients with no symptoms, this study further expands the current indications for CAC assessment to include symptomatic patients (5, 23, 42). Finally, this study suggests that patients already on statin may not benefit from this approach.

To the best of our knowledge, this is the first study evaluating the PCE and the Agatston score in a symptomatic, mixed Asian population. The prevalence of CAD in both symptomatic and asymptomatic Asian cohorts have been shown to be significantly different from Western cohorts typically used for derivation of risk scores (27, 29, 56). Consequently, CAC and other risk scores have shown reduced performance in these populations (57, 58). The ethnic admixture in Singapore covers predominantly Chinese, Malay and Indian populations. Because of its recent immigratory history, this cohort covers a genetically disproportionately broad representation of the Asian ethnogeography (36). The current study thus extends the use of the Agatston score to this group.

Secondarily, results from logistic regression demonstrate that population with non-zero value of CAC was statistically associated with MACE as compared to their CAC-free counterpart. While this adheres to the well-known 15-year warranty rule, it is a departure from a separate Asian study that suggests the lack of protective effect of being CAC-free in a Thai population (25). This is likely attributable difference in study population of study, with the study recruiting asymptomatic males almost exclusively. Furthermore, we noted that PCE score was statistically associated with MACE in the CAC-free cohort, but not their CAC > 0 counterpart, suggesting the possibility of a diminishing association between PCE score and MACE with the onset of CAC.

The findings of this study must be read within the boundaries of its limitations. Whilst statin status was known, duration, indication and statin dosage were not. This was a single center study with a relatively small cohort, and results must be interpreted as such. This current study was not the development of a new model, but rather a validation of an existing PCE model in an Asian cohort. Typically, both the PCE and CAC quantification have been validated in asymptomatic cohorts. Symptomatic cohorts, as in this study, are at higher risk of MACE. However, the current study necessitated the use of a risk stratification model as a basis of comparison for CAC in predicting MACE and thus utilized the PCE. This study is novel as it aims to explore the utility of CAC as an adjunct for risk stratification in symptomatic patients to ensure a more targeted patient selection for monitoring and intervention. Additionally, Singapore has a small population that is poorly understood with regards to ASCVD risk. This study is of clinical importance in understanding its healthcare burden. Future work includes development and subsequent validation of a more appropriate model for Singapore, using a larger cohort. There may have been incomplete patient follow up. As the loss to follow up may be assumed to occur equally across the study cohort, influence on the results may be minimal. This study did not evaluate performance between ethnicities, due to inadequate power. These findings cannot be extrapolated to the global Asian population, a diverse group comprising 60% of humankind (59). This study comprised a majority Chinese ethnicity component, known to have a lower CAD prevalence, and a relatively small proportion of South Asians, a group that has been shown to have a higher CVD risk (57, 60–62). Despite that, it is representative of Singapore’s ethnic composition, a unique singular cohort across three major Asian ethnicities (37). Whole genome sequencing uncovered 52 million novel variants with large genetic diversity within this population (36). As such, this study may have been underpowered to realize and reproduce the full incremental benefit of CAC assessment that has been shown in larger studies (7, 23, 46). This was a retrospective study with its accompanying biases. As an endpoint for this study, MACE included revascularization. Whilst this may sometimes be considered a “soft” clinical outcome, it was included as it nevertheless is associated with significant morbidity and mortality. To minimize bias, the study censored for revascularization performed before 180 days of the index scan.

In conclusion, in a symptomatic mixed Asian cohort, PCE assessment provided no significant discriminative value when compared to random chance in the overall cohort. However, the PCE score was associated with MACE in zero CAC subjects, but not in those with CAC > 0. Whilst the PCE provided poorer discriminatory performance in statin users, the Agatston score provided consistent discriminatory performance in both statin and non-statin users for MACE prediction.

## Data availability statement

The raw data supporting the conclusions of this article will be made available by the authors, without undue reservation.

## Ethics statement

The studies involving human participants were reviewed and approved by the SingHealth Centralised Institutional Review Board. Written informed consent for participation was not required for this study in accordance with the national legislation and the institutional requirements.

## Author contributions

LB: manuscript write-up and study supervision. JL: manuscript write-up, data formatting, and analysis. MK: manuscript formatting. YN: data formatting. SA’A, JH, WH, YY, DH, RN, ST, MA-M, MB, and LS: manuscript review. All authors contributed to the article and approved the submitted version.
